# Role of SiO_*x*_ in rice-husk-derived anodes for Li-ion batteries

**DOI:** 10.1038/s41598-022-04979-5

**Published:** 2022-01-19

**Authors:** Yusuke Abe, Masahiro Tomioka, Mahmudul Kabir, Seiji Kumagai

**Affiliations:** 1 Joint Research Center for Electric Architecture, Akita University, Tegatagakuen-machi 1-1, Akita, 010-8502 Japan; 2 Department of Mathematical Science and Electrical-Electronic-Computer Engineering, Akita University, Tegatagakuen-machi 1-1, Akita, 010-8502 Japan

**Keywords:** Batteries, Batteries, Batteries

## Abstract

The present study investigated the role of SiO_*x*_ in a rice-husk-derived C/SiO_*x*_ anode on the rate and cycling performance of a Li-ion battery. C/SiO_*x*_ active materials with different SiO_*x*_ contents (45, 24, and 5 mass%) were prepared from rice husk by heat treatment and immersion in NaOH solution. The C and SiO_*x*_ specific capacities were 375 and 475 mAh g^−1^, respectively. A stable anodic operation was achieved by pre-lithiating the C/SiO_*x*_ anode. Full-cells consisting of this anode and a Li(Ni_0.5_Co_0.2_Mn_0.3_)O_2_ cathode displayed high initial Coulombic efficiency (~ 85%) and high discharge specific capacity, indicating the maximum performance of the cathode (~ 150 mAh g^−1^). At increased current density, the higher the SiO_*x*_ content, the higher the specific capacity retention, suggesting that the time response of the reversible reaction of SiO_*x*_ with Li ions is faster than that of the C component. The full-cell with the highest SiO_*x*_ content exhibited the largest decrease in cell specific capacity during the cycle test. The structural decay caused by the volume expansion of SiO_*x*_ during Li-ion uptake and release degraded the cycling performance. Based on its high production yield and electrochemical benefits, degree of cycling performance degradation, and disadvantages of its removal, SiO_*x*_ is preferably retained for Li-ion battery anode applications.

## Introduction

Approximately 150 million tons of rice husk (RH) are generated annually and globally as agricultural waste^1^. RH is composed of approximately 80 mass% organic material (lignin, cellulose, hemicellulose, and others), and the rest are mainly silicon oxides (SiO_*x*_, 0 < *x* < 2)^2^. Rice, a silicicolous plant, adsorbs Si(OH)_4_ from the soil for growth and accumulates SiO_*x*_ in its husk^3^. RH is not preferred as a fuel because it produces a large amount of SiO_*x*_-derived ash during combustion. Thus, the utilization of RH has been limited to agricultural applications such as fertilizer additives, stockbreeding mats, and bed soil. On the other hand, RH, being intrinsically a composite of organic material and inorganic SiO_*x*_ itself, can be easily transformed into a C/SiO_*x*_ composite through simple heat treatment under inert conditions (carbonization).

Lithium-ion batteries (LIBs) have been used as power sources in various industrial applications, such as smartphones, industrial robots, electric vehicles, and grid-scale electricity storage systems. Graphite, hard carbon, Si, and SiO_*x*_ have been used as anodic active materials (AMs) in LIBs^4–10^. Anodic AMs operate using several Li storage mechanisms (insertion, conversion, and alloying reactions)^8,11–14^. Intercalation compounds, such as carbon materials, follow the intercalation mechanism, which facilitates Li-ion insertion (reduction) and extraction (oxidation)^11^. This mechanism provides a good conductive pathway, smooth Li-ion transport, and strong robustness during cyclic Li-ion insertion and extraction. Alloying compounds, such as Si and Sn, follow the alloying mechanism when they react with Li, which can lead to dramatically higher specific capacities for Li storage than those for the insertion mechanism^14^. High specific capacities are is also obtainable by the conversion mechanism associated with the reactions of transition metal oxides and Li. However, large irreversible capacities and positive anode potentials are induced by this mechanism compared to the other mechanisms.

Low electrode potential, high specific capacity for Li-ion uptake and release, good cycle stability, low toxicity, low cost, and natural abundance are required for anodic AMs. Graphite, which follows the insertion mechanism, is still mainly used in commercial LIBs because of its low and flat potential profile, low irreversible capacity, low cost, and accumulated experience. However, it has drawbacks, including a limited specific capacity of 372 mAh g^−1^, small margin for Li deposition or dendrite formation, and moderate cycle life^11,15^. Si, which follows the alloying mechanism, is anticipated to supersede graphite because of its very high specific capacity (4200 mAh g^−1^ at full Li alloying)^14^. However, it undergoes a large volume expansion during Li-ion uptake and release (~ 280%)^16^, causing the cracking of Si particles and thus, repeated formation of a solid-electrolyte interphase (SEI). Repeated SEI formation consumes Li ions in the electrolyte and increases the irreversible capacity of the Si anode. Moreover, cracking can induce the delamination of Si particles from the electrode, leading to capacity fading of the cell. Therefore, the sole use of Si in LIB anodes is still rare for commercial LIBs. However, the use of SiO_*x*_ has been an alternative countermeasure to increase the anode capacity of cells. High specific capacities of 1965 and 2043 mAh g^−1^ were obtained for SiO_2_ and SiO, respectively^7,17^. The reduction reaction of SiO_*x*_ with Li ions can produce lithium silicate (Li_*x*_SiO_*y*_) and lithium oxide (Li_2_O), leading to a smaller volume expansion at succeeding cycles of Li-ion uptake and release and thus, higher cycle stability^17^. However, SiO_*x*_ has a lower electrical conductivity than C and Si, requiring a high-level conductive agent (e.g., carbon black) to fabricate the anode. Thus, C/SiO_*x*_ composites have attracted much attention as anodic AMs for LIBs^18–21^.

For sustainable development goals, biomass-derived materials are viable options for next-generation LIB anodes that require high environmental compatibility. As mentioned above, C/SiO_*x*_ composites can be readily produced from RH via simple carbonization. RH-derived C/SiO_*x*_ composites have been investigated for application as LIB anodes. Ju et al. stated that RH-derived C/SiO_*x*_ exhibits a large irreversible capacity of 46.7% Coulombic efficiency (CE) at the initial Li-ion insertion and extraction^22^. Kumagai et al. also reported ~ 53% CE at the initial Li-ion uptake and release^17^. The large irreversible capacity of RH-derived C/SiO_*x*_ has been demonstrated elsewhere^15,20–23^. In addition, the Li-ion insertion and extraction properties of carbon materials produced by removing almost all SiO_*x*_ from carbonized RH were also evaluated^24,25^. Although SiO_*x*_ undergoes reduction reactions with Li ions and thereby increases the irreversible capacity, the CE of the carbon material is not high (~ 60%)^15,18^. SiO_*x*_ in RH acts as a template to produce micro- and mesoporous structures in the resultant carbon material^26^. The passivation of Li ions in the produced pores, as well as SEI formation on the increased surface area, is responsible for the large irreversible capacity^20,23^. To overcome the large irreversible capacity of RH-derived C/SiO_*x*_, a pre-lithiation process that provides sufficient Li ions to the anodic AMs can be a realistic choice^27–32^. Up to now, the Li-ion insertion and extraction properties of RH-derived C/SiO_*x*_ or C have been evaluated in the half-cell configuration where infinite Li ions can be provided by the Li metal electrode (counter and reference electrodes). However, for actual LIB full-cells, the large irreversible capacity of the anode has a decisive effect on the charge–discharge performance because a significant amount of Li ions can be consumed within and on the anode, and additional Li ions cannot be supplied from the outside. Although several studies have focused on the feasibility of RH-derived C/SiO_*x*_ as a LIB anodic AM, its electrochemical performance in a full-cell configuration has not been reported thus far. The role of SiO_*x*_ in LIB full-cell performance is also unknown.

In the present study, C/SiO_*x*_ was produced by heating RH under N_2_ and then immersing the carbonized RH in NaOH solution. By adjusting the immersion temperature and time, C/SiO_*x*_ composites with different SiO_*x*_ contents were prepared as LIB anodic AMs. The physical properties of the prepared samples were evaluated by thermogravimetric analysis, particle size analysis, and N_2_ adsorption–desorption porosimetry. CR2032-type half-cells and full-cells were assembled to evaluate the Li-ion insertion and extraction properties of C/SiO_*x*_. Pre-lithiation was performed prior to the LIB full-cell assembly. Full-cells with a pre-lithiated or pristine C/SiO_*x*_ anode and Li(Ni_0.5_Co_0.2_Mn_0.3_)O_2_ (NCM) cathode were assembled, and their rate and cycle performance were evaluated. Finally, the role of SiO_*x*_ in the C/SiO_*x*_ anode in the rate and cycle performance of the assembled full-cells was discussed. To the best of our knowledge, the performance of LIB full-cells with pre-lithiated C/SiO_*x*_ anodes has been evaluated for the first time in this study. Revealing the roles of the pre-lithiation process and SiO_*x*_ removal in the anode on the performance of LIB full-cells is scientifically novel and industrially and environmentally significant. The knowledge provided in this study is expected to promote the development of eco-friendly LIBs and the effective use of agricultural waste.

## Experimental methods

### Preparation of C/SiO_x_ AMs and their characterization

Raw RH (Akitakomachi rice) was provided by a rice farmer in Senboku City, Akita Prefecture, Japan with his permission and used as feedstock for RH-derived C/SiO_*x*_ AMs. The raw RH was dried at 100 °C for 10 h to determine the initial mass. Pre-carbonization was conducted in a tubular furnace by heating the raw RH at 600 °C for 1 h under N_2_ gas flow at 1 L min^−1^. The pre-carbonized RH was then immersed in a 1 mol L^−1^ NaOH solution under the following two conditions: (A) immersion temperature: 25 °C, immersion time: 22 h for half-elimination of SiO_*x*_ and (B) immersion temperature: 80 °C, immersion time: 10 h for complete elimination of SiO_*x*_. The mass ratio of pre-carbonized RH to the solution was 1:20. The pre-carbonized RH immersed in NaOH solution was rinsed with distilled water until the pH of the used rinse water was reduced to < 9. For the pre-carbonized RH that was not immersed in NaOH solution, the rinse consisting of distilled water was also prepared. All rinsed samples were dried at 100 °C for 10 h. For the main carbonization, they were heated to 1000 °C, and the temperature was maintained for 1 h in a similar manner to the pre-carbonization. Grains of the carbonized RH were pulverized using a planetary ball-milling machine with a zirconia vessel and balls (P-6, Fritch Japan Co., Ltd., Japan) at 400 rpm for 20 min, finally providing C/SiO_*x*_ AMs with different SiO_*x*_ contents.

The production mass yield was calculated based on the masses of the dried raw RH and prepared C/SiO_*x*_ AMs. The average particle diameters of the C/SiO_*x*_ AMs were measured using a laser diffraction particle size analyzer (SALD-200 V, Shimadzu Corp., Japan). The specific surface areas of the C/SiO_*x*_ AMs were measured using a gas adsorption analyzer (Autosorb-3B, Quantachrome Instruments Inc., USA). The Brunauer–Emmett–Teller specific surface area was calculated from the obtained nitrogen adsorption–desorption isotherms at − 196 °C. The total pore volume was measured using the volume of adsorbed nitrogen at a relative pressure close to 1 as reference. A thermogravimetry system (Thermo Plus EVO TG8120, Rigaku Corp., Japan) was used to heat the C/SiO_*x*_ AMs up to 850 °C under air flow (200 mL min^−1^). The SiO_*x*_ content was calculated from the mass of C/SiO_*x*_ at 140 and 850 °C. The physical properties of the three types of C/SiO_*x*_ AMs are summarized in Table 1. The C/SiO_*x*_ AMs are denoted as C/SiO_*x*_-45, C/SiO_*x*_-24, and C/SiO_*x*_-5 based on the SiO_*x*_ content. The production mass yield decreased with the SiO_*x*_ content and was reduced by ~ 14% owing to complete SiO_*x*_ removal. The particle sizes of the C/SiO_*x*_ AMs were adjusted to 4–5 µm. The removal of SiO_*x*_ increased the specific surface area and total pore volume, which is related to the decrease in the production mass yield.

**Table 1:** Physical properties of the C/SiO_*x*_ active materials.

	C/SiO_*x*_-45	C/SiO_*x*_-24	C/SiO_*x*_-5
Production mass yield (mass%)	35.5	25.7	21.1
Average particle diameter (µm)	4.6	4.2	4.5
Brunauer–Emmett–Teller specific surface area (m^2^ g^−1^)	44	125	193
Total pore volume (cm^3^ g^−1^)	0.07	0.17	0.23
SiO_*x*_ content (mass%)	44.6	23.6	4.8

### Electrode fabrication

To fabricate the anode, 80 mass% C/SiO_*x*_, 10 mass% conductive agent (acetylene black, Denka Co., Ltd., Japan), 7.5% sodium carboxymethyl cellulose (Cellogen 7A, DSK Co., Ltd., Japan), and 2.5 mass% styrene–butadiene rubber (TRD2001, JSR Corp., Japan) were dispersed in distilled water and mixed using a planetary centrifugal mixer (AR-100, Thinky Corp., Japan). The uniformly dispersed slurry was coated onto a copper foil (no surface treatment, *t*20 µm, Hohsen Corp., Japan) using an applicator and dried in an oven at 100 °C for > 6 h. The cathode for the full-cell assembly was also fabricated. A ternary lithium transition metal oxide (Li(Ni_0.5_Co_0.2_Mn_0.3_)O_2_, NCM, Beijing Easpring Material Technology Co., Ltd., China), acetylene black, and polyvinyl difluoride (KF polymer F #9130, Kureha Corp., Japan) were mixed at a mass ratio of 80:10:10 in *N*-methylpyrrolidone (Tokyo Chemical Industry Co., Ltd., Japan). The cathode slurry was coated onto an aluminum foil (no surface treatment, *t*20 µm, Hohsen Corp., Japan) and dried in an oven at 100 °C for > 6 h. Both dried electrodes were punched out into *ϕ*15-mm disks and dried at 140 °C under vacuum for 3 h. The loading masses of the AMs in the cathodes and anodes were respectively 5.57–5.61 and 2.15–2.31 mg cm^−2^; hence the electrode loading masses were 6.96–7.01 mg cm^−2^ for the NCM cathode and 2.69–2.89 mg cm^−2^ for the C/SiO_*x*_ anode. The coating thicknesses of the cathode and anode layers on the current collectors were 42–44 and 31–43 µm, respectively.

### Half-cell assembly and pre-lithiation of the C/SiO_*x*_ electrodes

The half-cell consisting of the C/SiO_*x*_ electrode and Li metal foil (*ϕ*15 mm, *t*0.2 mm, Honjo Metal Co., Ltd., Japan) was assembled using a CR2032 coin cell (Hohsen Corp., Japan) in an argon-filled glove box. The electrolyte used was 1 mol L^−1^ LiPF_6_ dispersed in 1:1 v/v% ethylene carbonate/diethyl carbonate (Kishida Chemical Co., Ltd., Japan). The two electrodes separated by a polypropylene separator (*ϕ*18 mm, 2500, Celgard LLC, USA) were installed in the coin cell after dipping the electrodes and separator in the electrolyte for ~ 5 s. A Li-ion insertion-extraction test was performed on the coin-type half-cells using a battery charge–discharge system (HJ1020mSD8, Hokuto Denko Corp., Japan). The electrode potential range was 0–2.5 V vs. Li^+^/Li, and the current density was 20 mA g_AM_^−1^, where g_AM_ is the unit of the mass of the C/SiO_*x*_ AM in the electrode. The Li-ion insertion-extraction process was repeated five times. The specific capacities of the C/SiO_*x*_ AMs were determined using the half-cell test results as the reference. The cycling stabilities of the C/SiO_*x*_ AMs were then evaluated by repeated (100-times) Li-ion insertion-extraction at a current density of 200 mA g_AM_^−1^ in a similar potential range.

### Assembly of the full-cells with pre-lithiated C/SiO_x_ electrode and their evaluation

**Figure 1:**
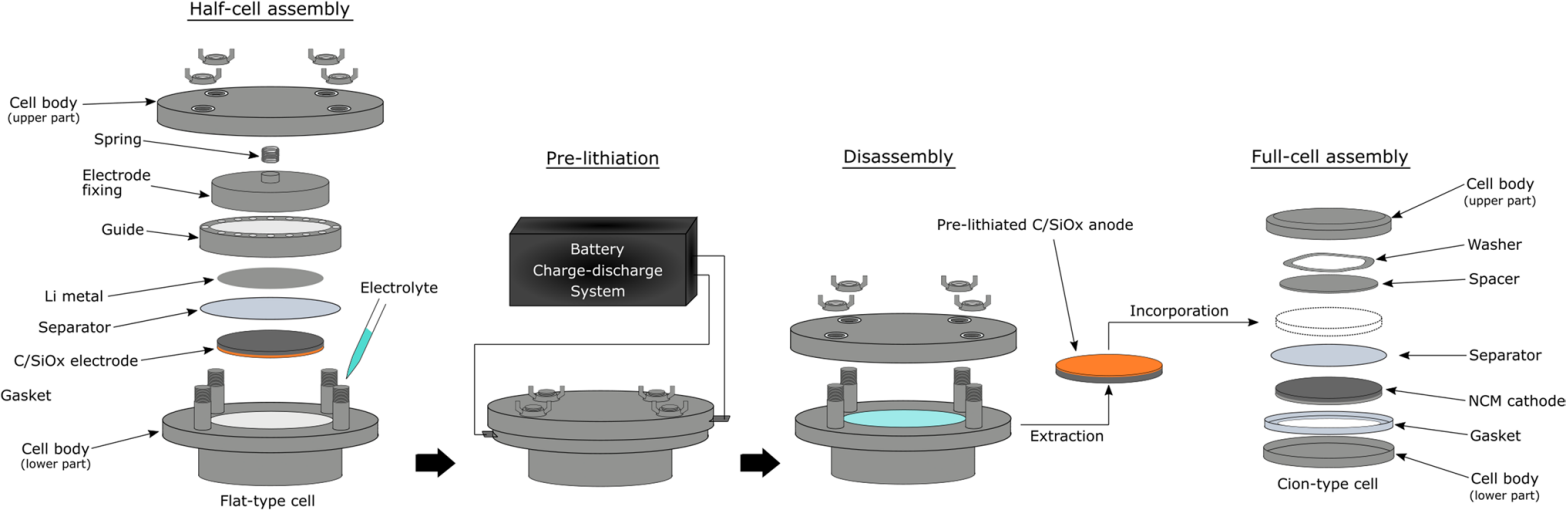
Pre-lithiation of the C/SiO_*x*_ anode and Li(Ni_0.5_Co_0.2_Mn_0.3_)O_2_ (NCM) + C/SiO_*x*_ full-cell assembly.

The specific capacity of the employed NCM AM was 150 mA g^−1^ based on the previous test results obtained using a half-cell configuration^28^. According to the specific capacity of the C/SiO_*x*_ AMs determined in the coin-type half-cell tests, the capacity ratio of the anode to the cathode in the full-cells was adjusted to 1.0 − 1.1. The pre-lithiation of the C/SiO_*x*_ electrode and NCM + C/SiO_*x*_ full-cell assembly is illustrated in Fig. 1. To prepare the pre-lithiated anode, a half-cell with the C/SiO_*x*_ electrode was separately assembled using an SUS304 stainless steel flat-type cell (HS Flat cell, Hosen Corp., Japan). The flat-type half-cell consisting of the C/SiO_*x*_ electrode, the above-mentioned Li metal foil, separator (*ϕ*23 mm), and electrolyte (1 mL) was assembled. The C/SiO_*x*_ electrode was pre-lithiated in an assembled flat-type half-cell. The potential of the C/SiO_*x*_ electrode decreased at a current density of 40 mA g_AM_^−1^, delivering Li ions to the C/SiO_*x*_ AM. The pre-lithiation sequence was stopped when the electrode potential reached 0 V vs. Li^+^/Li. Following pre-lithiation, the flat-type half-cell was introduced into an argon-filled glove box. The pre-lithiated C/SiO_*x*_ electrode was carefully extracted from the flat-type half-cell. Coin-type full-cells were then fabricated using the NCM cathode and pre-lithiated or unlithiated C/SiO_*x*_ anode in a similar manner to the coin-type half-cells.

The C-rate was employed to represent the current density of the full-cells. 1 C is defined as 150 mA g_CAM_^−1^, where g_CAM_ is the unit of the mass of the cathodic AM. The cell voltage range used for the charge–discharge sequences of the full-cells was 2.5–4.2 V. Charge–discharge cycling at 0.1 C was first performed three times for cell stabilization. Following cell stabilization, the rate performance of the full-cells was evaluated, during which the C-rate was increased stepwise from 0.1 to 10 C, and then decreased to 0.1 C. The charge–discharge process was repeated five times at each C-rate. The cycle performance of the full-cells was then evaluated by repeating the charge–discharge cycles at 2 C. The full-cell performance was represented by the cathode specific capacity (time integral of the cell current divided by the mass of cathode AM in units of mAh g_AM_^−1^).

## Results and discussion

### Li-ion insertion and extraction properties of C/SiO_x_ AMs

**Figure 2:**
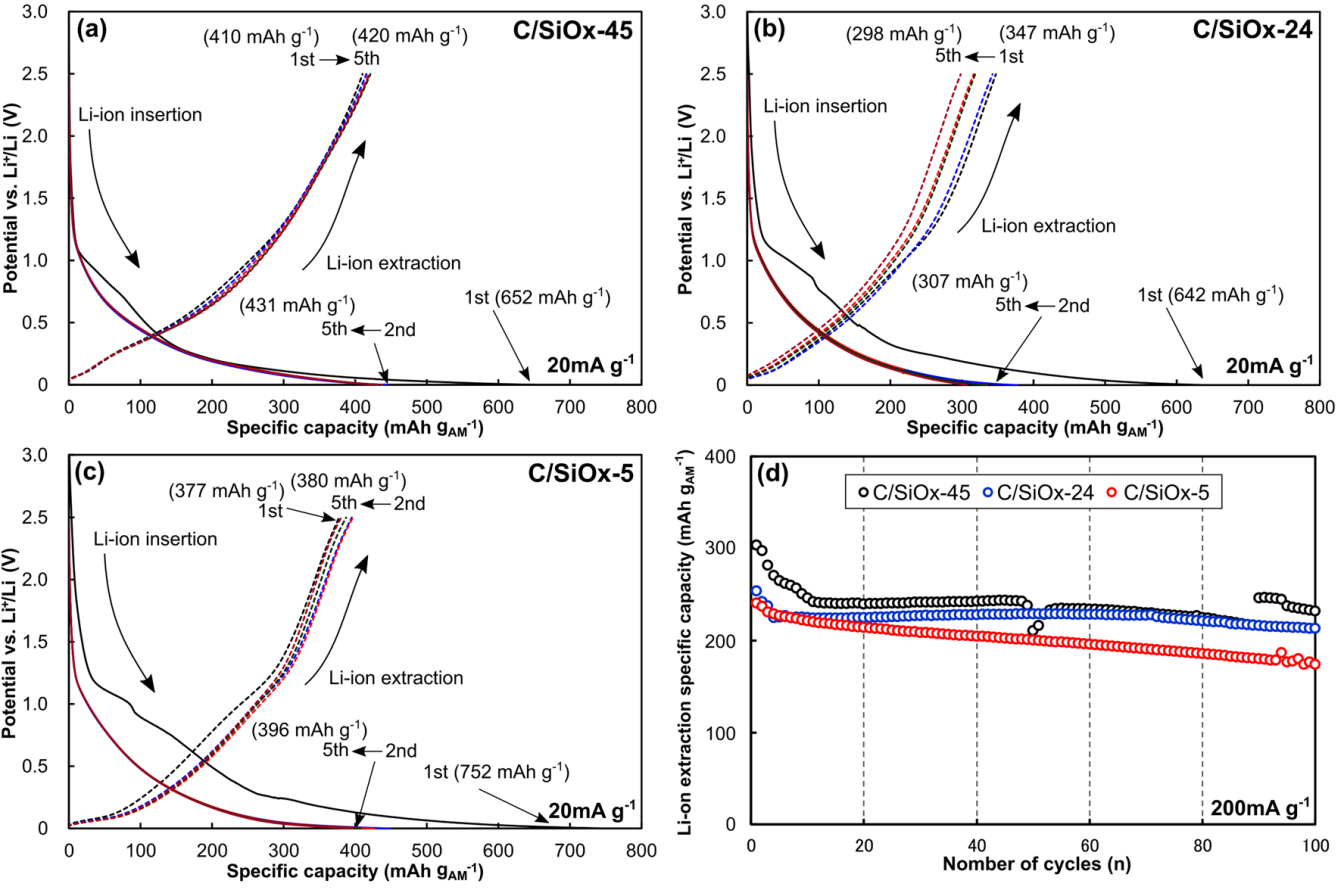
Initial Li-ion insertion and extraction properties of (**a**) C/SiO_*x*_-45, (**b**) C/SiO_*x*_-24, and (**c**) C/SiO_*x*_-5 in a coin-type half-cell configuration at 20 mA g_AM_^−1^, and (**d**) their cycling stabilities at 200 mA g_AM_^−1^ in the half-cell configuration in the 0–2.5 V (vs. Li^+^/Li) electrode potential range.

Figure 2 shows the initial Li-ion insertion and extraction properties of the C/SiO_*x*_ AMs at 20 mA g_AM_^−1^, and their cycling stabilities at 200 mA g_AM_^−1^ in the half-cell configuration. In the first cycle, C/SiO_*x*_-5 exhibited the highest Li-ion insertion specific capacity of 752 mAh g_AM_^−1^. A comparative Li-ion insertion specific capacity (~ 650 mAh g _AM_^−1^) was observed for C/SiO_*x*_-45 and C/SiO_*x*_-24. All C/SiO_*x*_ AMs displayed a Li-ion extraction specific capacity of < 410 mAh g_AM_^−1^, which was much lower than those for Li-ion insertion. The initial CEs of C/SiO_*x*_-45, C/SiO_*x*_-24, and C/SiO_*x*_-5 were 63.0%, 54.1%, and 50.2%, respectively, indicating that the initial CE decreases with decreasing SiO_*x*_ content. The CEs increased with the number of cycles. In the fifth cycle, C/SiO_*x*_-45 had the highest Li-ion extraction specific capacity (420 mAh g_AM_^−1^), while C/SiO_*x*_-24 had the lowest one (298 mAh g_AM_^−1^). C/SiO_*x*_-5 displayed a maximum specific capacity of 380 mAh g_AM_^−1^. The apparent specific capacity of the C/SiO_*x*_ AMs was determined as 400 mAh g_AM_^−1^. These results demonstrate that SiO_*x*_ is beneficial for increasing the specific capacity of C/SiO_*x*_ AMs. On the other hand, partial removal of SiO_*x*_ is detrimental to maintaining the specific capacity. Then, the cycling stabilities of C/SiO_*x*_ AMs were evaluated at 200 mA g_AM_^−1^. The specific capacities for Li-ion extraction from all C/SiO_*x*_ AMs decrease significantly within the initial 10 cycles in the half-cell configuration where ample Li-ions are delivered, after which they plateau during the remaining 100 cycles.

### Pre-lithiation of the C/SiO_x_ electrodes

**Figure 3:**
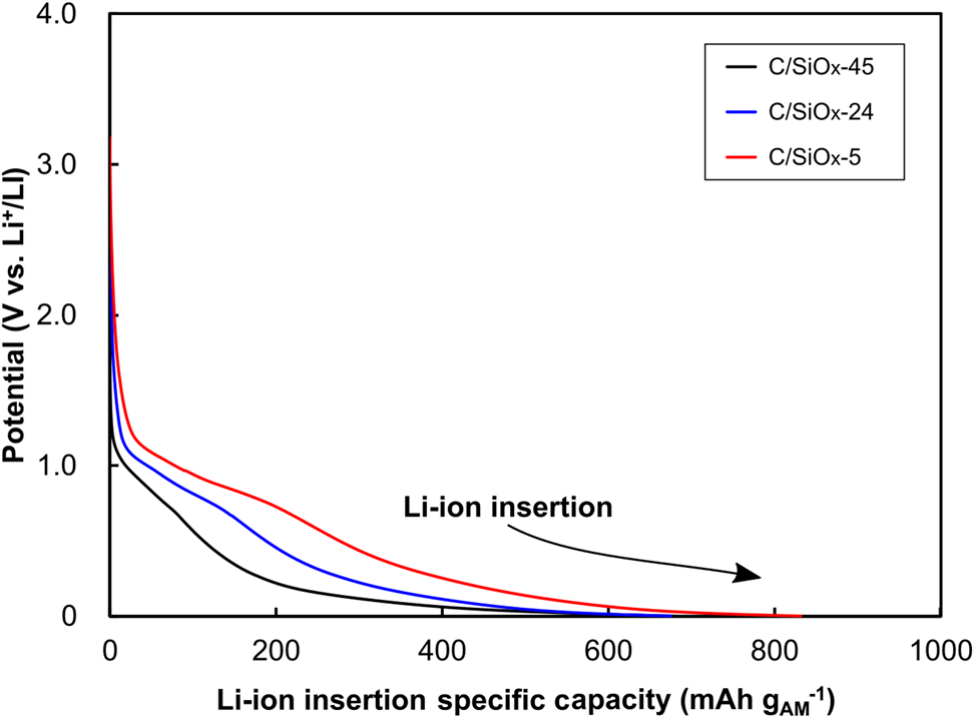
Li-ion insertion profiles of the C/SiO_*x*_ active materials during pre-lithiation.

The C/SiO_*x*_ AMs were pre-lithiated prior to full-cell assembly. The Li-ion insertion profiles of the C/SiO_*x*_ AMs in the flat-type half-cells are shown in Fig. 3. C/SiO_*x*_-45 and C/SiO_*x*_-24 displayed a similar Li-ion insertion specific capacity of ~ 680 mAh g_AM_^−1^, while C/SiO_*x*_-5 had a higher specific capacity of 832 mAh g_AM_^−1^. The C/SiO_*x*_ AMs exhibited different profiles at the first Li-ion insertion, and their potential levels decreased with increasing SiO_*x*_ content. The Li-ion insertion specific capacities of the C/SiO_*x*_ AMs obtained here were higher than those obtained using the coin-type half-cell, which can be attributed to the ample amount of electrolyte (1 mL) covering the electrodes.

### Performance of the NCM + C/SiO_x_ full-cells

Full-cells with the NCM cathode and pre-lithiated C/SiO_*x*_ anode were fabricated. A full-cell incorporating the unlithiated C/SiO_*x*_-45 anode (NCM + C/SiO_*x*_-45 without pre-lithiation) was also fabricated and evaluated. The charge–discharge specific capacities and CEs of the full-cells during the initial three cycles at 0.1 C are summarized in Table 2. In the first cycle, a large difference between the charge and discharge specific capacities (176 and 17 mAh g_CAM_^−1^, respectively) and a very low CE of 9.7% were observed for NCM + C/SiO_*x*_-45 without pre-lithiation. Even after the succeeding cycles, the discharge specific capacity was ~ 20 mAh g_CAM_^−1^. By pre-lithiating the C/SiO_*x*_ anodes, the CEs of the full-cells significantly increased up to ~ 85% in the first cycle. All full-cells with pre-lithiated anodes (pre-lithiated full-cells) exhibited stable charge–discharge profiles and very high CEs during the initial three cycles. In the 2nd cycle, the discharge specific capacities of the pre-lithiated full-cells reached the apparent specific capacity of the NCM cathode (150 mAh g_CAM_^−1^), indicating negligible loss of Li ions from the cathode.

**Table 2:** Charge (C_C_) and discharge (C_D_) specific capacities and Coulombic efficiencies (CE) of the Li(Ni_0.5_Co_0.2_Mn_0.3_)O_2_ (NCM) + C/SiO_*x*_ full-cells at the initial 3 cycles at 0.1 C.

Sample	1st			2nd			3rd		
	C_C_ (mAh g_CAM_^−1^)	C_D_ (mAh g_CAM_^−1^)	CE (%)	C_C_ (mAh g_CAM_^−1^)	C_D_ (mAh g_CAM_^−1^)	CE (%)	C_C_ (mAh g_CAM_^−1^)	C_D_ (mAh g_CAM_^−1^)	CE (%)
NCM + C/SiO_*x*_-45	158	135	85.4	149	147	98.7	151	149	98.7
NCM + C/SiO_*x*_-24	169	143	84.6	150	148	98.7	152	149	98.0
NCM + C/SiO_*x*_-5	168	143	85.1	154	152	98.7	156	153	98.1
NCM + C/SiO_*x*_-45 without pre-lithiation	176	17.0	9.7	30.1	19.6	65.1	28.6	20.9	73.0

**Figure 4:**
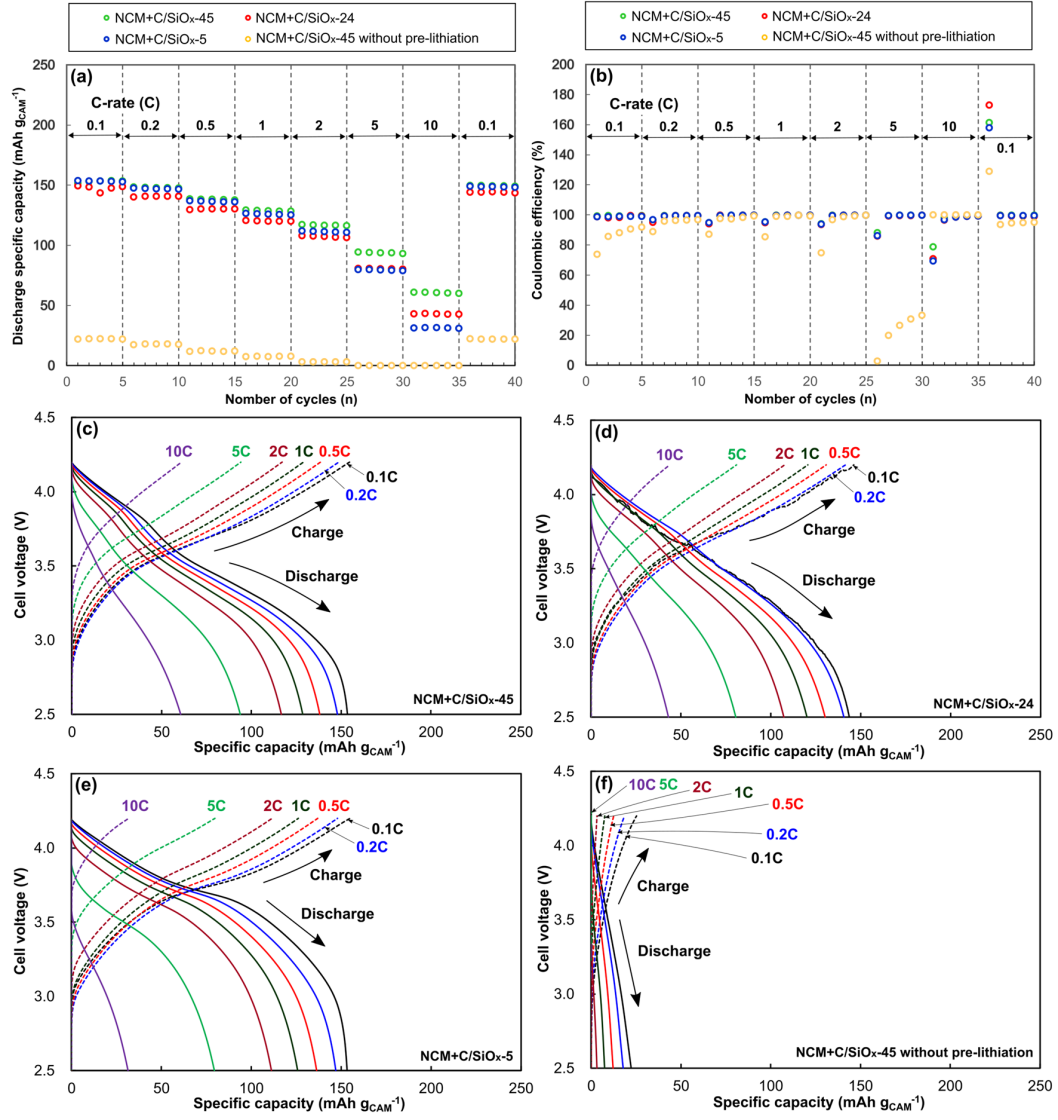
Rate performance of the Li(Ni_0.5_Co_0.2_Mn_0.3_)O_2_ (NCM) + C/SiO_*x*_ full-cells. (**a**) Discharge specific capacity and (**b**) Coulombic efficiency at different C-rates. Cell voltage-specific capacity profiles of the full-cells: (**c**) NCM + C/SiO_*x*_-45, (**d**) NCM + C/SiO_*x*_-24, (**e**) NCM + C/SiO_*x*_-5, and (**f**) NCM + C/SiO_*x*_-45 without pre-lithiation. The profiles of the 3rd cycle at each C-rate are shown. The cell voltage range was 2.5–4.2 V.

Following the initial three cycles, the rate performance of all full-cells was evaluated at a current density of 0.1–10 C and is shown in Fig. 4. Except for NCM + C/SiO_*x*_-45 without pre-lithiation, the full-cells displayed similar levels of discharge specific capacity up to 2 C. At higher current densities (5 and 10 C), a higher SiO_*x*_ content led to a higher discharge specific capacity. The discharge specific capacities of NCM + C/SiO_*x*_-45, NCM + C/SiO_*x*_-24, and NCM + C/SiO_*x*_-5 at 10 C were 60, 43, and 31 mAh g_CAM_^−1^, respectively. The CEs of the pre-lithiated full-cells were ~ 100%, except during the first cycle at each C-rate. The discharge specific capacity of NCM + C/SiO_*x*_-45 without pre-lithiation was much lower than those of the pre-lithiated full-cells throughout the rate test. The cell voltage-specific capacity profiles of NCM + C/SiO_*x*_-45 and NCM + C/SiO_*x*_-24 exhibited slope-like variations, while NCM + C/SiO_*x*_-5 maintained a higher cell voltage (allowing narrow plateau regions) than the others at low current densities (up to 2 C). Maintaining a high cell voltage increases the density of the energy delivered during the charge and discharge processes. At high current densities (> 2 C), the residual SiO_*x*_ in the anode was effective in sustaining the cell capacity.

**Figure 5:**
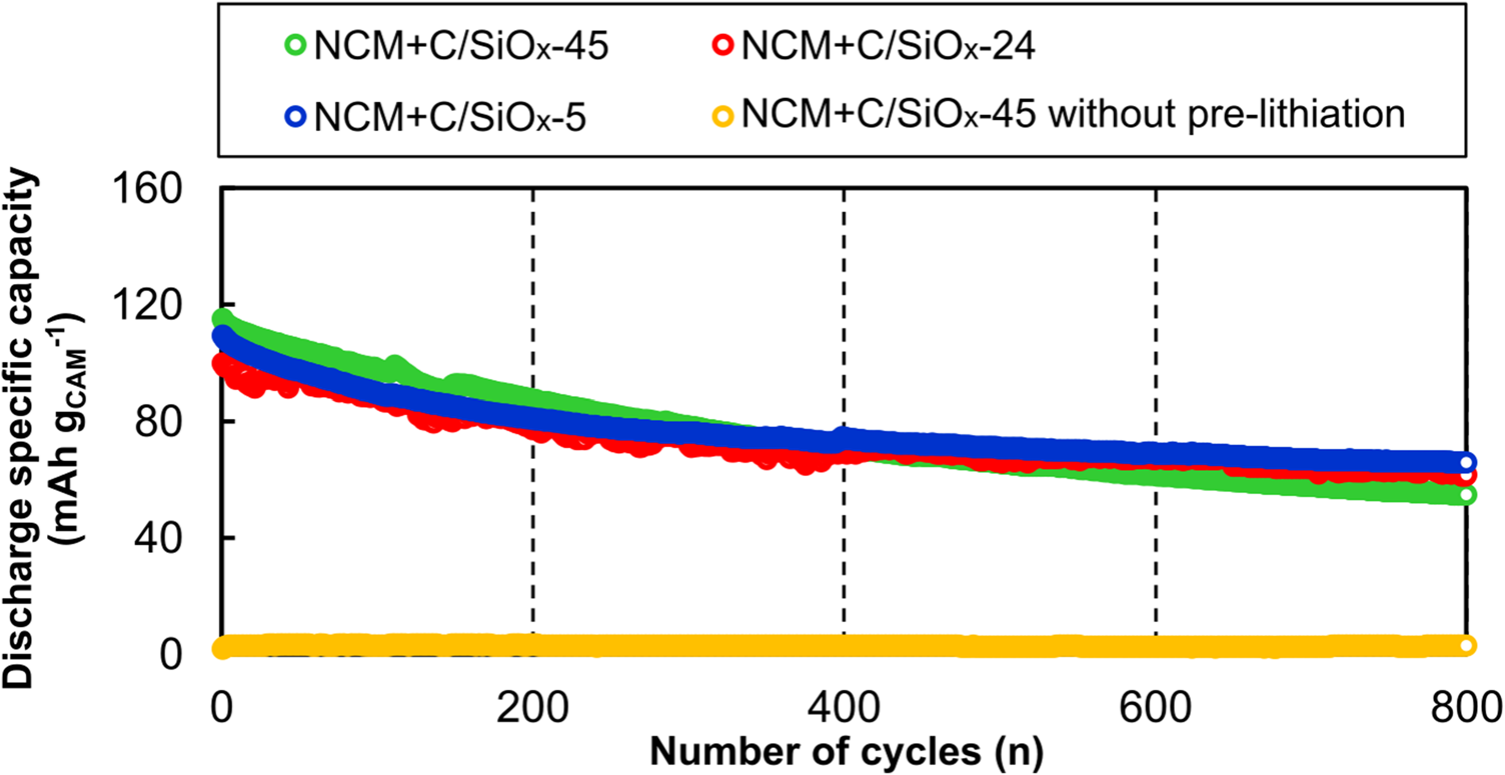
Cycling performance of the Li(Ni_0.5_Co_0.2_Mn_0.3_)O_2_ (NCM) + C/SiO_*x*_ full-cells. The cell voltage range was 2.5–4.2 V, and the charge–discharge current density was 2 C (300 mA g_CAM_^−1^).

The cycling performance of the full-cells at 2 C is shown in Fig. 5. The highest discharge specific capacity was displayed by NCM + C/SiO_*x*_-45 in the 1st cycle and NCM + C/SiO_*x*_-5 in the final cycle. All full-cells showed a gradual decrease in capacity with the number of cycles. The capacity retentions of NCM + C/SiO_*x*_-45, NCM + C/SiO_*x*_-24, and NCM + C/SiO_*x*_-5 after 800 cycles were 47.5%, 61.7%, and 60.7%, respectively. The presence of SiO_*x*_ in the anode led to a ~ 13% difference in the retention of the cell specific capacity. The largest decrease was observed for NCM + SiO_*x*_-45, which has the highest level of SiO_*x*_. The discharge specific capacity of NCM + C/SiO_*x*_-45 without pre-lithiation was negligible throughout the 800 cycles, suggesting that pre-lithiation is essential for RH-derived C/SiO_*x*_ AMs. Cycle aging was alleviated when the SiO_*x*_ in the anode was removed completely or partially. However, NCM + C/SiO_*x*_-24 showed an unstable variation in the discharge specific capacity up to the ~ 400th cycle.

### Effect of SiO_x_ in the anode on the full-cell performance

The number of oxygen atoms (*x*) in SiO_*x*_ in the RH carbonized at 1000 °C was found to be close to 2^17^. Under this assumption, the SiO_*x*_ in C/SiO_*x*_ AMs can react with Li ions according to the following chemical formulae [Eqs. (1) to (4)]^21^:

Reversible reactions (Li-ion insertion and extraction)

1 $$5{\mathrm{SiO}}_{2}+4{\mathrm{Li}}^{+}+4{\mathrm{e}}^{-}\leftrightarrow 2{\mathrm{Li}}_{2}{\mathrm{Si}}_{2}{\mathrm{O}}_{5}+\mathrm{Si}$$

2 $$\mathrm{Si}+{\mathrm{xLi}}^{+}+{\mathrm{xe}}^{-}\leftrightarrow {\mathrm{Li}}_{\mathrm{x}}\mathrm{Si}.$$

Irreversible reactions (Li-ion insertion only)

3 $${\mathrm{SiO}}_{2}+4{\mathrm{Li}}^{+}+4{\mathrm{e}}^{-}\to 2{\mathrm{Li}}_{2}\mathrm{O}+\mathrm{Si}$$

4 $$2{\mathrm{SiO}}_{2}+4{\mathrm{Li}}^{+}+4{\mathrm{e}}^{-}\to {\mathrm{Li}}_{4}{\mathrm{SiO}}_{4}+\mathrm{Si}.$$

In the half-cell configuration, the reversible (Li-ion extraction) capacities of C/SiO_*x*_-45 and C/SiO_*x*_-5 were 420 and 380 mAh g_AM_^−1^, respectively. These results indicate that the specific capacities of C and SiO_*x*_ were 375 and 475 mAh g_AM_^−1^, respectively. Based on the specific capacity of SiO_2_ (1965 mAh g^−1^)^7^, the irreversible reactions in Eqs. (3) and (4) dominate. However, approximately one-quarter of SiO_*x*_ was responsible for the reversible specific capacity for Li-ion insertion and extraction via Eqs. (1) and (2). On the other hand, C/SiO_*x*_-24 had the lowest specific capacity of 298 mAh g_AM_^−1^. Sodium silicates such as Na_2_SiO_3_ are likely produced during SiO_*x*_ leaching, i.e*.*, immersion in NaOH solution^20^. They are inactive for Li-ion uptake and release, which reduced the specific capacity of C/SiO_*x*_-24. C/SiO_*x*_ AMs with a lower SiO_*x*_ content exhibited a lower initial CE. The removal of SiO_*x*_ from C/SiO_*x*_ AMs increased the specific surface area (44 m^2^ g^−1^ for C/SiO_*x*_-45 and 193 m^2^ g^−1^ for C/SiO_*x*_-5) because micro- and mesopores were produced at the trace of SiO_*x*_^26^. As pores develop in RH-derived anodic AMs, more Li ions can be passivated within the pores^20,23^. Thus, the lower the SiO_*x*_ content, the lower the initial CE.

The physical properties of the C/SiO_*x*_ AMs affected the rate and cycle performance of the NCM + C/SiO_*x*_ full-cells. Pre-lithiation was demonstrated to be required for LIBs with RH-derived anodic AMs. Owing to pre-lithiation, all full-cells displayed a high discharge specific capacity of ~ 150 mAh g_CAM_^−1^ at 0.1 C. At the increased C-rates of 5 and 10 C, the discharge specific capacity was more retained when C/SiO_*x*_-45 was used as the anode, indicating that the time response of the reversible reactions of SiO_*x*_ with Li ions [Eqs. (1) and (2)] is higher than that of the remaining C component. During the cycle test, the full-cell with the higher SiO_*x*_ content could not retain the initial cell specific capacity, and unstable capacity variation was observed for NCM + C/SiO_*x*_-24. Although the SiO_*x*_ component in C/SiO_*x*_ AM provided a higher time rate for Li-ion insertion and extraction, the uptake and release of Li ions in SiO_*x*_ [Eqs. (1) and (2)] seem to cause a large volume expansion, thereby leading to structural decay in the anode. The unstable variation of NCM + C/SiO_*x*_-24 could be explained by the formation of inactive sodium silicates during SiO_*x*_ leaching.

The textural and electrochemical properties of RH-derived AMs intended for use in LIB anodes are summarized in Table 3. The reversible specific capacities of RH-derived AMs produced in the present study are lower than those reported in the relevant literature. However, C/SiO_*x*_-45, produced through two-step carbonization, exhibited the highest CE (63.0%) among all AMs, and the CEs of C/SiO_*x*_-24 and C/SiO_*x*_-5 are also high compared to those of the other AMs. A trade-off relationship between specific capacity and CE seems to be closely related to porosity, which is represented by the specific surface area or the total pore volume of the AM. Pre-lithiation was shown to be essential for the RH-derived AMs produced here when employed in a LIB full-cell, suggesting that pre-lithiation is necessary for all anodic AMs produced from RH for stable operation in LIB full-cells.

**Table 3:** Comparing the textural and electrochemical properties of RH-derived AMs intended for the in LIB anodes.

Sample	Preparation notes	Content of Si-based materials (mass%)	SA (m^2^ g^−1^)	V_total_ (cm^3^ g^−1^)	CE_Initial_ (%)	C_R_ (mAh g^−1^)	Ref
C/SiO_*x*_-45	Carbonization, rinse, and carbonization	45 (SiO_*x*_)	44	0.07	63.0	420 @20 mA g^−1^	This work
C/SiO_*x*_-24	Carbonization, immersion in NaOH solution at 25 °C, rinse, and carbonization	24 (SiO_*x*_)	125	0.17	54.1	298 @20 mA g^−1^	This work
C/SiO_*x*_-5	Carbonization, immersion in NaOH solution at 80 °C, rinse, and carbonization	5 (SiO_*x*_)	193	0.23	50.2	380 @20 mA g^−1^	This work
Nano-Si/C	Addition of Mg into RH, carbonization, and neutralization using HCl solution	18 (Si)	–	–	59.6	560 @100 mA g^−1^	18
Activated carbon	Carbonization, chemical activation using NaOH, and neutralization	–	2176	0.91	45.8	608 @0.2 C	20
C/SiO_2_	Addition of ZnCl into RH, carbonization, and neutralization using HCl solution	57 (SiO_2_)	1191	0.40	–	~ 750 @200 mA g^−1^	21
SiO_*x*_/C	Carbonization	–	–	–	46.7	582 @100 mA g^−1^	22
C/SiO_2_	Formic acid treatment, hydrothermal processing, carbonization, and SiO_*x*_ removal with ammonium hydrogen fluoride	–	243	0.41	49.8	~ 400 @75 mA g^−1^	23
Porous C	Immersion in H_2_SO_4_ solution, hydrothermal processing, carbonization, and immersion in NaOH solution, and neutralization	–	332	–	43.8	~ 757 @74 mA g^−1^	33

SA: specific surface area, V_total_: total pore volume, CE_Initial_: initial Coulombic efficiency, C_R_: reversible specific capacity.

In the present study, the partial removal of SiO_*x*_ using the NaOH solution likely produced a sodium silicate byproduct, which is inactive for Li-ion uptake and release. This byproduct formation rather destabilized the cycle performance of the full-cell. It was shown that the specific capacity of the SiO_*x*_ component was 100 mAh g_AM_^−1^ higher than that of the C component in the RH-derived anodic AM. In addition, the time response of the SiO_*x*_ component for Li-ion uptake and release was also higher, thereby delivering the highest rate performance to the NCM + C/SiO_*x*_ full-cells. However, the SiO_*x*_ component reduced the specific capacity retention of the full-cells during the cycle test. Judging from the minor difference in the specific capacity retention (~ 13% in 800 cycles), significant effort to completely remove SiO_*x*_, excellent rate performance provided by SiO_*x*_, and formation of sodium silicate byproduct, it is not strongly necessary to remove SiO_*x*_ from the RH-derived AM intended for LIB anodes. The important task of the present study was to develop sustainable and eco-friendly LIB anode materials. In this sense, it is preferrable to preserve SiO_*x*_ to avoid using a strong alkaline solution for SiO_*x*_ leaching and strong acid for neutralization. The high production yield due to SiO_*x*_ preservation (~ 14 mass% higher than that for SiO_*x*_ removal) is also beneficial for industrial applications.

## Conclusions

RH-derived C/SiO_*x*_ AMs with 45, 24, and 5 mass% SiO_*x*_ were prepared by heat treatment and immersion in a NaOH solution. C/SiO_*x*_ AMs that were pre-carbonized at 600 °C, then subjected to SiO_*x*_ leaching, and finally carbonized at 1000 °C were evaluated as Li-ion battery anodes in half-cell and full-cell configurations. The specific capacities of the C and SiO_*x*_ components were 375 and 475 mAh g^−1^, respectively, indicating that only about one-quarter of SiO_*x*_ was responsible for the reversible specific capacity for Li-ion insertion and extraction. C/SiO_*x*_ AMs with a lower SiO_*x*_ content exhibited a lower initial CE for Li-ion insertion and extraction because of Li passivation within the pores developed at the trace of the removed SiO_*x*_. Full-cells with a NCM cathode and C/SiO_*x*_ anode were assembled. Owing to the low initial CE (< 65%), pre-lithiation was essential to attain a stable anodic operation for the C/SiO_*x*_ AMs. All full-cells exhibited a high initial CE (~ 85%) and high discharge specific capacity (~ 150 mAh g_CAM_^−1^) at 0.1 C. At the increased C-rates of 5 and 10 C, a higher SiO_*x*_ content in the full-cell led to a higher cell specific capacity retention, revealing that the reversible reactions of SiO_*x*_ with Li ions are faster than those of the C component. The full-cell with C/SiO_*x*_-45 exhibited the largest decrease in cell specific capacity (47.5% retention) during the cycle test, while the other full-cells exhibited ~ 60% retention. All results indicated that the SiO_*x*_ component in the RH-derived AMs enhances the specific capacity and rate performance, while inducing structural decay owing to the volume expansion of SiO_*x*_. Considering the higher production yield, enhanced specific capacity and rate stability due to SiO_*x*_, acceptable cycle performance degradation, and operational effort, cost, and eco-friendliness of SiO_*x*_ removal, the preservation of SiO_*x*_ is recommended when carbonized RHs are utilized as LIB anodes.

## References

[1] Chen R, Congress SSC, Cai G, Duan W, Liu S (2021). Sustainable utilization of biomass waste-rice husk ash as a new solidified material of soil in geotechnical engineering: A review. Constr. Build. Mater..

[2] Kumagai S (2019). Lithium-ion capacitor using rice husk-derived cathode and anode active materials adapted to uncontrolled full-pre-lithiation. J. Power Sources.

[3] Jung DS, Ryou M-H, Sung YJ, Park SB, Choi JW (2013). Recycling rice husks for high-capacity lithium battery anodes. Proc. Natl. Acad. Sci. U. S. A..

[4] Azam MA, Safie NE, Ahmad AS, Yuza NA, Zulkifli NSA (2021). Recent advances of silicon, carbon composites and tin oxide as new anode materials for lithium-ion battery: A comprehensive review. J. Energy Storage.

[5] Song BF, Dhanabalan A, Biswal SL (2020). Evaluating the capacity ratio and prelithiation strategies for extending cyclability in porous silicon composite anodes and lithium iron phosphate cathodes for high capacity lithium-ion batteries. J. Energy Storage.

[6] Abe Y, Kumagai S (2018). Effect of negative/positive capacity ratio on the rate and cycling performances of LiFePO_4_/graphite lithium-ion batteries. J. Energy Storage.

[7] Shobukawa H, Alvarado J, Yang Y, Meng YS (2017). Electrochemical performance and interfacial investigation on Si composite anode for lithium ion batteries in full cell. J. Power Sources.

[8] Kim HJ (2016). Controlled prelithiation of silicon monoxide for high performance lithium-ion rechargeable full cells. Nano Lett..

[9] Yang X (2015). The contradiction between the half-cell and full-battery evaluations on the tungsten-coating LiNi_0.5_Co_0.2_Mn_0.3_O_2_ cathode. Electrochim. Acta.

[10] Loeffler N (2014). Performance of LiNi_1/3_Mn_1/3_Co_1/3_O_2_/graphite batteries based on aqueous binder. J. Power Sources.

[11] Liu Q (2016). Understanding undesirable anode lithium plating issues in lithium-ion batteries. RSC Adv..

[12] Wang C (2016). A robust strategy for crafting monodisperse Li_4_Ti_5_O_12_ nanospheres as superior rate anode for lithium ion batteries. Nano Energy.

[13] Yi T (2021). Approaching high-performance lithium storage materials by constructing hierarchical CoNiO_2_@CeO_2_ nanosheets. Energy Environ. Mater..

[14] Hassoun J, Derrien G, Panero S, Scrosati B (2008). A nanostructured Sn-C composite lithium battery electrode with unique stability and high electrochemical performance. Adv. Mater..

[15] Dawei L (2021). Adjusting ash content of char to enhance lithium storage performance of rice husk-based SiO_2_/C. J. Alloys Compd..

[16] Eguchi T, Sawada K, Tomioka M, Kumagai S (2021). Energy density maximization of Li-ion capacitor using highly porous activated carbon cathode and micrometer-sized Si anode. Electrochim. Acta.

[17] Kumagai S, Abe Y, Tomioka M, Kabir M (2021). Suitable binder for Li-ion battery anode produced from rice husk. Sci. Rep..

[18] Chu H, Wu Q, Huang J (2018). Rice husk derived silicon/carbon and silica/carbon nanocomposites as anodic materials for lithium-ion batteries. Colloids Surf. A Physicochem. Eng. Aspects.

[19] Wang SE, Jang I-S, Kang YC, Chun J, Jung D-S (2021). Residual silica removal and nanopore generation on industrial waste silicon using ammonium fluoride and its application to lithium-ion battery anodes. Chem. Eng. J..

[20] Kaifeng Y, Li J, Qi H, Liang C (2018). High-capacity activated carbon anode material for lithium-ion batteries prepared from rice husk by a facile method. Diam. Relat. Mater..

[21] Cui J (2017). High surface area C/SiO_2_ composites from rice husks as a high-performance anode for lithium ion batteries. Powder Technol..

[22] Ju Y (2016). SiO_x_/C composite from rice husks as an anode material for lithium-ion batteries. Electrochim. Acta.

[23] Wang L, Schnepp Z, Titirici MM (2013). Rice husk-derived carbon anodes for lithium ion batteries. J. Mater. Chem. A.

[24] Rybarczyk MK (2019). Hard carbon derived from rice husk as low cost negative electrodes in Na-ion batteries. J. Energy Chem..

[25] Fan X (2017). Fe_3_O_4_/rice husk-based maco-/mesoporous carbon bone nanocomposite as superior high-rate anode for lithium ion battery. J. Solid State Electrochem..

[26] Kumagai S, Sato M, Tashima D (2013). Electrical double-layer capacitance of micro- and mesoporous activated carbon prepared from rice husk and beet sugar. Electrochim. Acta.

[27] Jiao L (2018). An advanced lithium ion battery based on a high quality graphitic graphene anode and a Li[Ni_0.6_Co_0.2_Mn_0.2_]O_2_ cathode. Electrochim. Acta.

[28] Abe Y, Saito T, Kumagai S (2018). Effect of prelithiation process for hard carbon negative electrode on the rate and cycling behaviors of lithium-ion batteries. Batteries.

[29] Son IH (2017). Graphene balls for lithium rechargeable batteries with fast charging and high volumetric energy densities. Nat. Commun..

[30] Libich J, Máca J, Vondrák J, Čech O, Sedlaříková M (2017). Irreversible capacity and rate-capability properties of lithium-ion negative electrode based on natural graphite. J. Energy Storage.

[31] Zhang J, Liu X, Wang J, Shi J, Shi Z (2016). Different types of pre-lithiated hard carbon as negative electrode material for lithium-ion capacitors. Electrochim. Acta.

[32] Wang Z (2014). Application of stabilized lithium metal powder (SLMP^®^) in graphite anode—A high efficient prelithiation method for lithium-ion batteries. J. Power Sources.

[33] Yu K (2019). Preparation of porous carbon anode materials for lithium-ion battery from rice husk. Mater. Lett..

